# Tinospora cordifolia (Guduchi/Giloy)-Induced Liver Injury: A Case Review

**DOI:** 10.7759/cureus.39793

**Published:** 2023-05-31

**Authors:** Ikenna Nnamani, Oluwaremilekun Tolu-Akinnawo, Rabira R Dufera, Akintomiwa Akintunde, Benedict Maliakkal

**Affiliations:** 1 Internal Medicine, Meharry Medical College, Nashville, USA; 2 Gastroenterology and Hepatology, Nashville General Hospital, Nashville, USA

**Keywords:** autoimmunity, transaminases, drug-induced liver injury (dili), herb-induced liver injury (hili), giloy, guduchi, tinosporium cordifolia, hepatotoxicity

## Abstract

*Tinospora cordifolia* (Guduchi/Giloy) is a relatively common herbal supplement whose use has recently become prominent in Southeast Asia. It was promoted to the public in India as an immunity booster, especially against the novel COVID-19. There have been reports, mostly from India, of an association between Guduchi/Giloy and liver injury. We present a 50-year-old female with a history of Hashimoto thyroiditis, who presented with abdominal discomfort and nausea of two weeks duration, which coincided with starting HistaEze^TM^ supplement containing *Tinospora cordifolia*. The vital signs upon presentation showed no significant abnormalities. Labs were significant for severely elevated transaminases; however, viral panels, autoimmune serologies, and imaging studies were unremarkable. Roussel Uclaf causality assessment method (RUCAM) score was at 6, which was indicative of probable drug/herb-induced liver injury. HistaEze^TM^ was discontinued, and the patient took a three-day course of oral steroids with significant interval improvement in clinical status, as evidenced by progressive normalization of the transaminases level. The transaminases decreased by greater than 50% within two weeks of discontinuation and trended back to baseline within three months. This case highlights the worldwide availability and use of *Tinospora cordifolia*, which can cause liver injury that appears to be idiosyncratic and possibly immune-mediated. Further research on the precise mechanism of its hepatotoxicity is warranted.

## Introduction

Dietary and herbal supplements are known to be significant contributors to acute liver injury in the United States, accounting for about 15.5% of drug-induced liver injury (DILI), and are also a major cause of fulminant hepatitis in the Western world [1,2]. It’s known that drugs and herbal-induced liver injury (HILI) replicate features of acute or chronic liver diseases, which are associated with genetic and environmental factors that modify the metabolism and elimination of drugs [2]. Drug-induced liver injury has been associated with liver failure resulting in death or liver transplantation in about 10% of cases [3]. In the United States, the use of complementary and alternative medical (CAM) therapies like herbal and dietary supplements has increased, especially in women and people under 65 years [4]. *Tinospora cordifolia*, commonly described as heart-leaved moonseed (also known as Guduchi in Sanskrit or Giloy in Hindi), is an Ayurvedic herbal supplement used as an immune booster mostly in India, especially during the COVID-19 pandemic [5]. It has recently been shown to cause acute hepatitis [6]. Here, we present one of the first reported cases of *Tinospora cordifolia*-induced liver injury in the United States.

## Case presentation

A 50-year-old female with a past medical history of Hashimoto thyroiditis on levothyroxine replacement therapy presented with mild abdominal discomfort of two weeks duration. Abdominal pain was constant, graded 3-4/10 in severity, located in the right upper quadrant and epigastrium region, non-radiating, and had no known aggravating or relieving factors. The pain was associated with generalized fatigue, loss of appetite, nausea, and rare vomiting. Four to five weeks prior to presentation, the patient started taking 1 tablet of HistaEze^TM^, which contains 900 mg of *Tinospora cordifolia* extract (stem)/Giloy supplement for immune support against COVID-19 infection. Other than the reported symptoms above, the patient denied weight changes, regurgitation, heartburn, jaundice, skin rash, pruritus, change in stool or urine color, recent travel, exposure to blood or body fluids, needle stick injuries, or change in bowel habits. The patient reported a family history of autoimmune diseases; however, she was unsure of the specific type. She denied a history of smoking or alcohol intake and had no recreational drug use. Active medications at presentation include levothyroxine sodium, progesterone, estradiol patch, and HistaEze^TM^ supplement. 

On presentation to the gastrointestinal/liver clinic, her vitals were unremarkable. Physical examination noted mild discomfort to palpation in the right upper quadrant without rebound tenderness or guarding. Labs were notable for elevated alanine transaminase (ALT) 1336 IU/L and aspartate aminotransferase (AST) 375 IU/L (reference range: ALT 6-50 IU/L; AST 8-46 IU/L). Alkaline phosphatase (ALP) was 57 IU/L (reference range: 45-117 IU/L), which was normal. Likewise, albumin, bilirubin, thyroid function tests, and the coagulation panel were all within normal limits. The hepatotropic and non-hepatotropic viral panels, autoimmune serologies, and imaging were also unremarkable. The Roussel Uclaf causality assessment model (RUCAM) score was 6, indicating probable drug-induced liver injury. 

**Figure 1:**
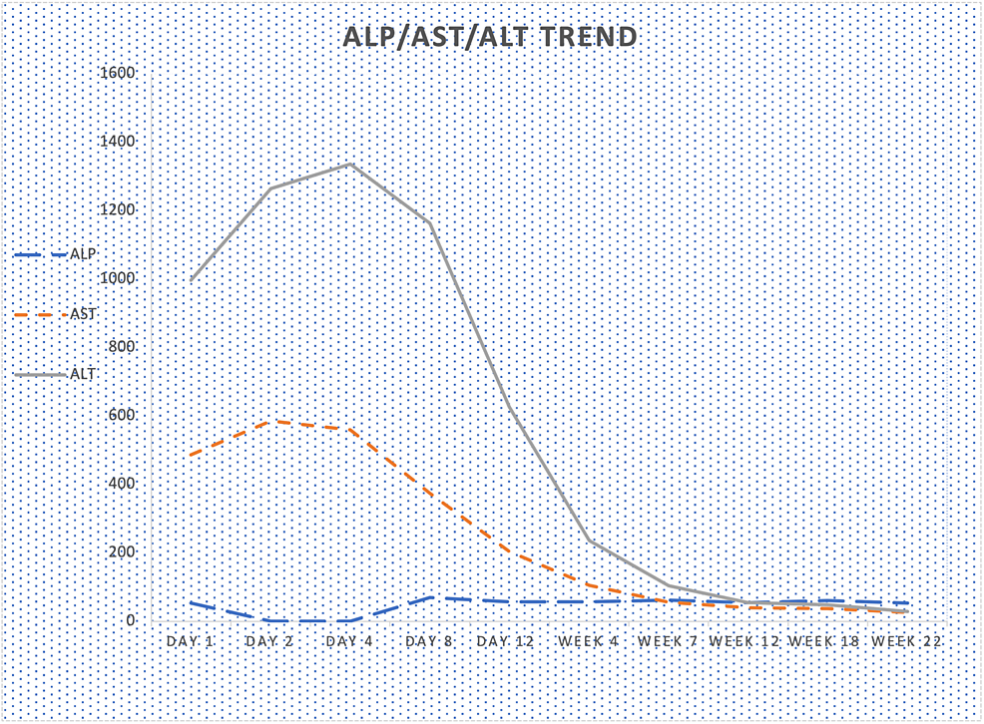
Trending of transaminases and ALP ALP: Alkaline phosphatase, AST: Aspartate aminotransferase, ALT: Alanine transaminase,

The patient was managed as an outpatient in the gastroenterology clinic, advised to stop taking HistaEze^TM^, and counseled on liberal oral fluid intake. The patient received a three-day course of prednisone 40 mg daily during the first week of the presentation, with interval improvement in symptoms noted. Table 1 below highlights the trend of transaminases and ALP over six months. Liver enzymes were monitored closely and started trending down significantly (ALT/AST 626/204 IU/L) within two weeks after discontinuation of the herbal supplement (Figure 1) and returned to baseline within three months (ALT/AST 48/38 IU/L).

**Table 1:** Laboratory results BUN: Blood urea nitrogen

Labs/Reference range	Laboratory trend (days/weeks)									
	Day 1	Day 2	Day 4	Day 8	Day 12	Week 4	Week 7	Week 12	Week 18	Week 22
Glucose (65–99 mg/dl)				97	84	73	92	93	82	
BUN (7–25 mg/dl)				13	11	15	15	13	12	
Creatinine (0.5–1.03 mg/dl)				0.62	0.68	0.74	0.59	0.59	0.56	
Sodium (135–146 mmol/l)				139	136	138	137	139	139	
Potassium (3.5–5.3 mmol/l)				4.4	4.5	4.4	4.4	4.2	4.2	
Chloride (98–110 mmol/l)				107	101	105	105	106	106	
Bicarbonate (20–32 mmol/l)				28	27	26	25	23	27	
Calcium (8.6–10.4 mg/dl)				9.5	9.4	9.2	9.3	9.6	9.3	
Total protein (6.1–8.1 g/dl)				7.7	6.8	6.9	6.7	7.0	6.7	6.3
Albumin (3.6–5.1 g/dl)				4.0	4.3	4.4	4.3	4.5	4.5	4.1
Bilirubin (0.2–1.2 mg/dl)	0.9			0.6	0.6	1.0	0.6	0.6	1.0	0.7
Alkaline phosphatase (37–153 U/L)	53			70	57	57	62	55	61	53
Aspartate aminotransferase (10–35 U/L)	487	586	560	375	204	105	56	40	38	26
Alanine transaminase (6–29 U/L)	997	1264	1336	1163	626	236	104	55	48	29
White blood count (3.8–10.8 thousand/UL)				6.3	5.2	4.5	6.2	5.8		
Hemoglobin concentration (11.7–15.5 (g/dl)				14.5	13.9	14.2	14.4	16.4		
Hematocrit (35%–45%)				43.7	41.0	41.5	41.3	46.9		
Platelets (140–400 thousand/UL)				240	232	222	206	233		

## Discussion

Herbal supplements, although used quite frequently for improving health, have been associated with damage to the body, especially the liver. However, many patients do not fully volunteer this information as they feel it is harmless and can be misdiagnosed as acute viral hepatitis, autoimmune hepatitis, or alcoholic liver disease [7]. Its effect and extent of liver damage can be accessed with blood tests, liver biopsy, and histologic analysis and can be further classified into hepatotoxic and cholestatic injuries [8]. Drug-induced liver injury may occur in a predictable or idiosyncratic pattern [5,9], which could be metabolic or immune-mediated. 

HistaEze^TM^ contains quercetin 600 mg, stinging nettle (Urtica dioica) 600 mg, and sodium bicarbonate in addition to *Tinospora cordifolia* extract (stem). There are no case reports of liver injury from quercetin or Urtica dioica which have both been widely used all over the world for many years. Hence, we suspect that the liver injury is from *Tinospora cordifolia* extract [10]. 

Although the precise pathophysiology of *Tinospora cordifolia*-induced liver injury remains unclear, its mechanism of toxicity appears to be an idiosyncratic liver injury, or immunomodulatory mechanism, or the unveiling of subtle autoimmune hepatitis. Autoimmunity has been thought to play a role as liver histology shows some similarities with atypical autoimmune features like hepatic plasma-lymphocytic infiltrations but without explicit features of autoimmune hepatitis and negative anti-smooth antibodies [5]. Hence, associations with other autoimmune diseases like type 1 diabetes mellitus, hypothyroidism, lupus, etc. may need to be further studied. Human leukocyte antigen (HLA) typing of the patients affected may also be helpful.

Herb-induced liver injury shares similarities with DILI regarding manifestations, which may depend on frequency, dosage, duration, and associated risk factors like autoimmunity and chronic liver disease. The median time from intake of *Tinospora cordifolia* to symptom onset is about 46 days [5]. The patient may be asymptomatic at presentation; however, symptoms could range from fatigue, nausea, vomiting, appetite loss, and abdominal pain to jaundice, pruritus, dark urine, and pale stool [5,7]. The severity of symptomatology may be attributed to many risk factors like baseline chronic liver disease, history of autoimmune disease, dosage, duration, and drug-to-drug/herb interactions [5]. 

Before diagnosing HILI, it is imperative to thoroughly rule out other causes of liver injury by obtaining a liver and coagulation panel, viral hepatitis panel, autoimmune serologies, and imaging. The RUCAM score, which is used as a grading system for clinical diagnosis of DILI, could be utilized for HILI as well: <1 excluded; 1-3 unlikely; 3-5 possible; 6-8 probable, and >8 highly probable [11]. A liver biopsy could be performed for histological evaluation, especially when the diagnosis remains unclear. 

Treatment entails discontinuing the offending agent and monitoring liver enzymes weekly or biweekly for a gradual decline in transaminase levels. Steroid therapy may be considered as there are some features of drug-induced autoimmune hepatitis with this herbal supplement and reports of faster improvement of liver labs and spontaneous resolution [12]. The prognosis for HILI is good if the offending substance is discontinued early. However, prognosis in the setting of underlying liver disease or comorbidities remains unclear and needs further prospective studies [7].

## Conclusions

This case highlights a case of *Tinospora cordifolia*-induced liver injury, whose use has become prevalent since the COVID-19 pandemic. A high level of suspicion is needed due to its close similarities to other forms of liver injury. Early identification and discontinuation of the offending medication provide critical diagnostic and prognostic value. Further studies are needed to define the prognosis of patients with underlying liver disease and co-morbidities. There is also a critical need to raise public awareness of the dangers of herbal supplements, which are not well-regulated, unlike allopathic medications.
